# Response to letter: “Re: discharge NIHSS scores are predictive of poor 3-month outcomes in patients with acute ischemic stroke receiving intravenous thrombolysis”

**DOI:** 10.1080/07853890.2026.2627095

**Published:** 2026-02-14

**Authors:** Longhai Zhu, Yan Qin, Tingting Kang, Yaoyu Ying, Yongjun Cao, Jijun Shi

**Affiliations:** ^a^ Department of Neurology, The Second Affiliated Hospital of Soochow University, Suzhou, China; ^b^ Department of Neurology, Nuclear Industry 417 Hospital, Xi’an, China; ^c^ Department of Medical Affairs, The Second Affiliated Hospital of Soochow University, Suzhou, China; ^d^ Clinical Research Center of Neurological Disease, The Second Affiliated Hospital of Soochow University, Suzhou, China

Dear Editor

We thank Dr. Yang Duan for the thoughtful comments on our article, “Discharge NIHSS Scores Are Predictive of Poor 3-month Outcomes in Patients with Acute Ischemic Stroke Receiving Intravenous Thrombolysis” (Ann Med. 2026;58(1):2610555). We appreciate the engagement with our work and the opportunity to clarify and extend several points.

We agree that the discharge NIHSS score is a composite measure, integrating treatment response, in-hospital recovery, and complications. While baseline and 24-hour scores provide important snapshots of neurological severity, the discharge assessment captures the overall neurological status at a pivotal transition point from acute care to rehabilitation. Its predictive superiority in our analysis likely reflects this integrative nature, summarizing the culmination of early recovery dynamics. We acknowledge that framing discharge NIHSS within a causal or mediation framework could enhance mechanistic understanding, and this represents a valuable direction for future research. Furthermore, future studies with larger sample sizes are warranted to clarify the predictive value of longitudinal NIHSS trajectories, rather than single time-point scores, for 3-month functional outcomes.

Our findings further reinforce the importance of early aggressive treatment and prompt rehabilitation initiation. The strong predictive value of discharge NIHSS scores highlights that patients who do not achieve adequate early neurological recovery may benefit from more intensive or targeted rehabilitation strategies, underscoring the need for a seamless transition from acute care to structured rehabilitation programs to optimize long-term functional outcomes.

Regarding the optimal cutoff value (NIHSS ≥4.5) derived from the Youden index, we acknowledge that non-integer thresholds may limit direct clinical interpretability. In practice, adjacent integer values (e.g. NIHSS ≥4 or ≥5) offer clinically applicable alternatives, maintaining robust discriminative performance in our cohort. The primary aim of reporting a precise statistical optimum was to establish a methodological benchmark; subsequent validation incorporating decision-curve analysis would help translate this threshold into clinically actionable guidance.

Finally, we agree that the discharge NIHSS score may serve as a bridge between acute care and recovery planning. Identifying patients with residual deficits (e.g. NIHSS ≥4–5) may highlight the need for more tailored post-acute interventions. The suggestion to integrate this with interdisciplinary, human-centered approaches, including art-based therapies to support rehabilitation engagement, is thought-provoking and aligns with emerging holistic paradigms in stroke care.

In summary, we are grateful to Dr. Duan [1] for highlighting these constructive considerations. Our study emphasizes the clinical utility of the discharge NIHSS score as an integrative prognostic tool at a critical transition point in patient care. We believe that addressing these insightful points will not only refine prognostic models but also catalyze research aimed at personalizing recovery pathways, emphasizing the continuum from early active treatment to comprehensive rehabilitation, ultimately improving outcomes for stroke survivors.

## Supplementary Material

Title page.docx

## Data Availability

There is no data associated with this research.
