# Supplementary material for: Response to letter: “Re: discharge NIHSS scores are predictive of poor 3-month outcomes in patients with acute ischemic stroke receiving intravenous thrombolysis”
Source: Ann Med. 2026 Feb 14;58(1):2627095. doi: 10.1080/07853890.2026.2627095 (PMC12912225; doi:10.1080/07853890.2026.2627095)
Supplement: Title page.docx [file IANN_A_2627095_SM2344.docx]

**Response to Letter: “Re: Discharge NIHSS Scores Are Predictive of Poor 3-month Outcomes in Patients with Acute Ischemic Stroke Receiving Intravenous Thrombolysis”**

Longhai Zhu^1#^, BD, Yan Qin^1#^, BD, Tingting Kang^1, 2#^, BD, Yaoyu Ying^3^, MB, Yongjun Cao^1,4*^, MD,PhD, Jijun Shi^1,4*^, MD

^1^Department of Neurology, the Second Affiliated Hospital of Soochow University, Suzhou, Jiangsu Province, 215004, China

^2^Department of Neurology, Nuclear Industry 417 Hospital, Xi’an, Shanxi Province, 710600, China

^3^Department of Medical Affairs, the Second Affiliated Hospital of Soochow University, Suzhou, Jiangsu Province, 215004, China

^4^Clinical Research Center of Neurological Disease, The Second Affiliated Hospital of Soochow University, Suzhou, Jiangsu Province, 215004, China

**Running title:** Discharge NIHSS score predicts poor outcomes

***Correspondence to**:

*Jijun Shi

Department of Neurology, The Second Affiliated Hospital of Soochow University, Suzhou, 215004, China

Tel. : +86 512 67783689; Fax: +86 512 67784303

E-mail: shijijun2008@126.com

ORCID: 0000-0003-3852-3993

*Yongjun Cao

Department of Neurology, The Second Affiliated Hospital of Soochow University, Suzhou, 215004, China

Tel.: +86 512 67783662; Fax: +86 512 67784303

E-mail: yongjuncao@126.com.

ORCID: 0000-0002-8135-8914

^#^Longhai Zhu, Yan Qin, and Tingting Kang contributed equally.

^*^Jijun Shi and Yongjun Cao jointly supervised this work.

**Keywords** NIHSS score; Ischemic stroke; Intravenous thrombolysis; Prognosis; Functional outcome
